# Performance of the First-Trimester Cervical Consistency Index to Predict Preterm Birth

**DOI:** 10.3390/jcm13133906

**Published:** 2024-07-03

**Authors:** Carlos H. Becerra-Mojica, Miguel A. Parra-Saavedra, Ruth A. Martínez-Vega, Luis A. Díaz-Martínez, Raigam J. Martínez-Portilla, Johnatan Torres-Torres, Bladimiro Rincon-Orozco

**Affiliations:** 1 School of Medicine, Universidad Industrial de Santander, Bucaramanga 680002, Colombia; ladimar@uis.edu.co; 2 Maternal-Fetal Medicine Unit, Hospital Universitario de Santander, Bucaramanga 680002, Colombia; 3 Centro de Atención Materno-Fetal INUTERO, Floridablanca 681004, Colombia; 4 Departamento Ginecologia y Obstetricia, Universidad Libre, Barranquilla 080003, Colombia; miguelparra51@hotmail.com; 5 Escuela de Medicina, Universidad de Santander, Bucaramanga 680003, Colombia; rutharam@yahoo.com; 6 Clinical Research Division, National Institute of Perinatology, Mexico City 11000, Mexico; raifet@hotmail.com (R.J.M.-P.); torresmmf@gmail.com (J.T.-T.)

**Keywords:** preterm birth, cervical consistency index, cervical length, preterm birth prediction

## Abstract

**Background/Objectives**: Preterm birth (PTB) remains a significant global health challenge. Previous attempts to predict preterm birth in the first trimester using cervical length have been contradictory. The cervical consistency index (CCI) was introduced to quantify early cervical changes and has shown promise across various clinical scenarios in the mid-trimester, though testing in the first trimester is lacking. This study aims to assess the cervical consistency index performance in predicting preterm birth during the first trimester of pregnancy. **Methods**: In this prospective cohort study, focused exclusively on research, women with singleton pregnancies, both with and without a history of spontaneous preterm birth (sPTB), were included. The primary outcome was sPTB before 37 weeks, with a secondary outcome of sPTB before 34 weeks. CCI measurements were taken between 11^+0^ to 13^+6^ weeks of gestation. Receiver operating characteristic (ROC) curves were generated, and sensitivity and specificity were calculated for the optimal cut-off and for the 5th, 10th, and 15th percentile. Intraobserver and interobserver agreements were assessed using the intraclass correlation coefficient (ICC). **Results:** Among the 667 patients analyzed, the rates of sPTB before 37 and 34 weeks were 9.2% (61/667) and 1.8% (12/667), respectively. The detection rates (DRs) for CCI predicting PTB before 37 and 34 weeks were 19.7% (12/61) and 33.3% (4/12). Negative predictive values were 91.8% (546/595) and 98.7% (588/596), while the areas under the curve (AUC) for sPTB before 37 and 34 weeks were 0.62 (95% CI: 0.54–0.69) and 0.80 (95% CI: 0.71–0.89), respectively. Of the 61 patients with preterm birth, 13 (21.3%) had a preterm birth history; in this group, the CCI percentile 10th identified 39% (5/13). Intraobserver ICC was 0.862 (95% CI: 0.769–0.920), and interobserver ICC was 0.833 (95% CI: 0.722–0.902). **Conclusions**: This study suggests that utilizing CCI in the first trimester of pregnancy could serve as a valuable tool for predicting preterm birth before 34 weeks of gestation, demonstrating robust intraobserver and interobserver reliability.

## 1. Introduction

Preterm birth (PTB), defined as childbirth occurring before 37 weeks of gestation, remains a significant global health challenge [1]. According to WHO estimates, approximately 15 million PTB cases occur annually [2]. Colombia has the highest PTB rates (10%) in Latin America, as reported by UNICEF [3,4]. This alarming statistic underscores PTB’s status as a leading cause of neonatal and under-five mortality, contributing to the death of approximately one million neonates annually due to PTB-related complications [5]. Numerous investigations have been conducted to predict and prevent PTB, resulting in substantial evidence supporting the use of cervical length measurement between 18 and 22 weeks of gestation. This currently stands as the standard method to identify pregnant women at risk of PTB, identifying 28% of pregnant women who will deliver between 34 to 37 weeks using a cut-off of 25 mm and a false positive rate of 10% [6]. Through a tailored approach, the detection rate of spontaneous preterm birth (sPTB) before 37 weeks using cervical length can be substantially enhanced, reaching as high as 50% [7].

To address the need to search for better predictive tools for sPTB, Parra et al. introduced the Cervical Consistency Index (CCI) as an ultrasound measurement for identifying accelerated cervical softening in 2011 [8]. The CCI quantifies the change of the anteroposterior diameter of the uterine cervix after deformation induced with the transvaginal probe by the operator. Notably, CCI has displayed good performance in various clinical scenarios, including low-risk pregnant women [9], high-risk pregnant women [10], and twin pregnancies [11], outperforming the cervical length measurements typically conducted during mid-trimester evaluations. However, CCI requires specific training to perform adequate measurements. Despite its promise, a recent review showed that more evidence is needed about CCI performance in order to achieve robust results in different clinical scenarios, especially in a prospective cohort developed for research purposes [12].

In an effort to tackle the PTB rates and to look for better prediction in the first trimester of pregnancy, we sought to assess the predictive capability of CCI measured during the first trimester for the prediction of sPTB before 37 weeks of gestation.

## 2. Materials and Methods

### 2.1. Study Design and Participants

This is a prospective cohort of singleton pregnant women between 11^+0^ to 13^+6^ weeks of gestation recruited from November 2019 to September 2022. Participants were recruited from the maternal–fetal units of two health institutions in Bucaramanga, Colombia, namely Hospital Universitario de Santander and Centro de Atencion Maternal-Fetal INUTERO. The Committee on Ethics and Research from the Universidad Industrial de Santander and participating centers approved the study (CEINCI: Act No. 17, 17 October 2019. Project code 254). All pregnant women signed informed consent for participation in this study. Criteria for inclusion were pregnant women between 11^+0^ to 13^+6^ weeks with a singleton pregnancy, including previous sPTB and nulliparous women. Criteria for exclusion were women with history of cervical surgery or Mullerian anomalies, PTB due to fetal or maternal indications (non-spontaneous), or pregnancies that ended before 22 weeks of gestation.

### 2.2. Recruitment

All eligible women who attended the 11^+0^ to 13^+6^ screening were invited to participate. Data were obtained from pregnant women using a standard survey at enrollment, including age, obstetric history, history of preterm birth, and cervical procedures. Anthropometric data were obtained before the ultrasound evaluation to determine typical risk calculations for chromosomal abnormalities and risk of preeclampsia.

### 2.3. Ultrasound Evaluation

Highly skilled maternal–fetal specialists, each with over five years of experience in 11^+0^ to 13^+6^ ultrasound screening scans, conducted the examinations. The medical team underwent rigorous training to master the CCI measurement technique, led by Dr. Parra-Saavedra, the creator of the method. Following the acquisition of ten practice images, feedback was provided, and a predetermined format was applied to assess competence. The assessment process involved registering a transabdominal image in the longitudinal middle plane of the uterus and cervix to evaluate cervical canal orientation. Subsequently, a transvaginal ultrasound, utilizing a 4–9 MHz endocavitary probe (Voluson E6, S8 General Electric, Milwaukee, MI, USA), was conducted.

**Figure 1:**
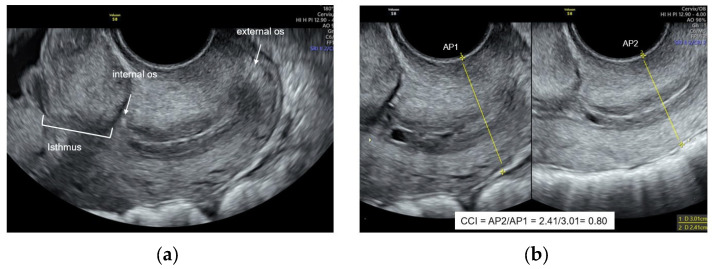
Cervical measurements. (**a**) Ultrasound image characteristics for first-trimester cervical length measurement. (**b**) Anteroposterior diameter before pressure (AP1) and anteroposterior diameter after probe pressure (AP2) to obtain the cervical consistency index (CCI).

As described elsewhere [13,14], cervical length measurement was executed by tracing a line between the internal and external os as reference points, avoiding the isthmus while ensuring proper orientation. For the CCI measurement, the double-screen image was activated, maintaining the same plane, and after freezing the left image, pressure with the probe was applied in an anteroposterior direction until the minimum diameter was obtained. AP diameters at the mid-third of the cervix were recorded for both images, and a second measurement of AP diameters was obtained to estimate intraobserver agreement (Figure 1). The CCI was calculated by dividing the AP diameter after probe pressure by the pre-pressure diameter, generating a value between 0 and 1, which was duly registered. Another team member, ensuring operator blinding, obtained the CCI value posteriorly.

Interobserver agreement was assessed by two independent operators in a subset of patients, with image evaluations and measurements conducted by experts MPS and CB. Importantly, the results of CCI measurements did not influence clinical decisions, and operators remained blinded to both CCI values and patient outcomes, maintaining scientific integrity throughout the study and avoiding bias. None of the patients received progesterone prescriptions, ensuring unbiased assessment and objective evaluation.

### 2.4. Follow Up

After the baseline evaluation, we performed a monthly telephone call to monitor the pregnancy’s evolution until delivery. A record was kept with the date of delivery, the delivery route, the delivery characteristics; and if it was spontaneous or was indicated by fetal or maternal conditions, the reason was also recorded. These data were obtained from the medical records of each patient.

### 2.5. Data Collection

Clinical data were stored in a password-protected, web-based electronic database, REDCap, with the de-identification capability to protect patient information. After extraction from the ultrasound machine, the deidentified ultrasound images were stored in a repository and linked through the unique assigned code.

### 2.6. Outcomes

The primary outcome was the occurrence of preterm birth, defined as childbirth before 37 weeks of gestation. The secondary outcome was preterm birth before 34 weeks of gestation.

### 2.7. Statistical Analysis

Mean and standard deviation were used for normally distributed data while the median and interquartile range (IQR) was used for non-parametric data. Categoric data were expressed as proportions and percentages. ROC curves were generated for the CCI, for PTB before 37 weeks and for PTB before 34 weeks. The sensitivity, specificity, positive (PPV) and negative (NPV) predictive values, and positive (LR+) and negative (LR−) likelihood ratios were calculated for the best cut-off obtained from the ROC curves and for intended additional cut-offs as the percentile 5, 10, and 15. The association between PTB, CCI, CL, and history of PTB was performed by multiple logistic regression analysis, while the performance was assessed using sensitivity, specificity, PPV and NPV, LR+ and LR−, and area under the curve (AUC). In addition, the intraobserver and the interobserver reliability were evaluated with the intraclass correlation coefficient (ICC) (two-way random effect model). Intraobserver agreement was calculated as the difference between two CCI measurements by the same observer, and the interobserver agreement as the difference between the CCI measurements obtained by different observers. The magnitude of the differences was estimated as described by Bland–Altman [15]. (StataCorp. 2020, Stata Statistical Software: Release 16. StataCorp LLC: College Station, TX, USA).

## 3. Results

### 3.1. Description of the Cohort and Characteristics of the Study Population

**Figure 2:**
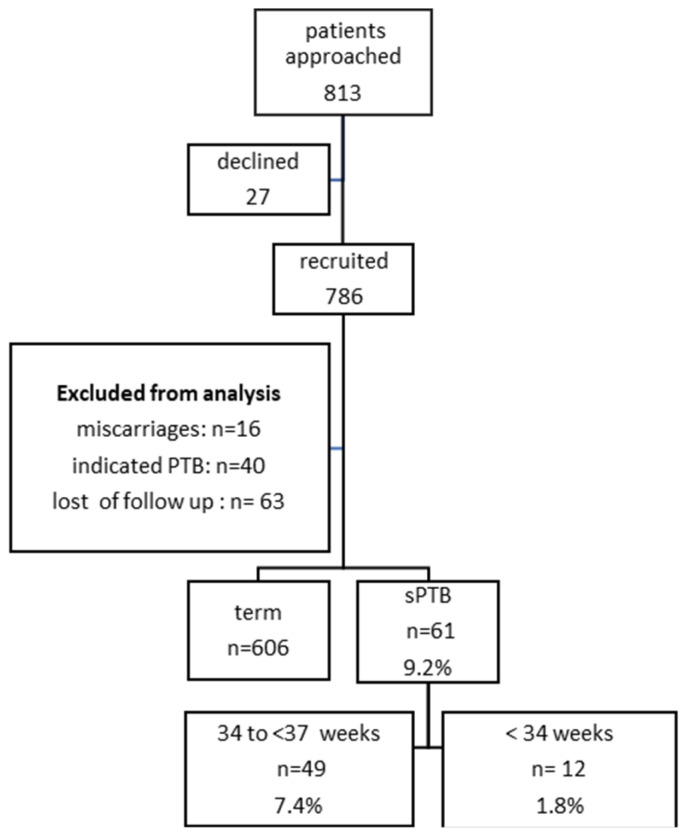
Patient flowchart.

Among the 786 pregnant women initially recruited, *45* were excluded due to miscarriages (16), iatrogenic preterm birth (40), and cases with incomplete perinatal outcomes (63) (Figure 2).

For the 667 women left for analysis, the median maternal age was 28 years. History of PTB was present in 8.1% (54/667) of the population, 55% (371/667) had a previous term birth, and the rest were nulliparous. The rate of sPTB < 37 and <34 weeks was 9.2% (61/667) and 1.8% (12/667), respectively. Women delivering < 37 weeks had a higher prevalence of history of PTB compared to patients delivering at term (21.3% vs. 6.8%: *p* < 0.001) and a lower CCI (0.80 vs. 0.83; *p* = 0.003) at first trimester, with no significant difference in CL (35 mm vs. 35 mm; *p* = 0.845) (Table 1).

Supplementary Table S1 contains the clinical and demographic characteristics of the entire cohort.

### 3.2. Cervical Consistency Index as Predictor of sPTB

A first-trimester CCI < 10th percentile (CCI < 0.74) has 20% sensitivity and 90% specificity for the prediction of sPTB < 37 weeks. Meanwhile, the same percentile has a 33% sensitivity and 90% specificity for sPTB < 34 weeks. The positive and negative LRs for the 10th percentile were 2.05 and 0.89 for sPTB < 37 weeks, and 3.35 and 0.74 for sPTB < 34 weeks. The cut-off point with the best LR+ for sPTB < 37 weeks was 0.70 CCI with a 9.8% detection rate (DR) at a 4% FPR and positive and negative LRs of 3.4 and 0.87, respectively. The best cut-off point for sPTB < 34 weeks was 0.69 CCI with a DR of 17% at a 3.7% FPR and 4.53 and 0.87 positive and negative LRs (Table 2).

### 3.3. Cervical Consistency Index as Predictor of sPTB According to History of sPTB

Using the same 10th percentile of CCI (CCI < 0.74) as a cut-off point, we divided the women in the study into two groups, namely those with preterm birth histories and those without. CCI sensitivity was better in women with previous sPTB compared to those without history of sPTB. However, specificity was lower in women with history of sPTB. The best sensitivity was found among women with history of sPTB that delivered before 34 weeks (67%), while the highest specificity was the same for women without history of sPTB overall (Table 3).

### 3.4. Association between sPTB, CCI, Cervical Length, and History of PTB

In a multiple regression analysis, the two independent variables associated with sPTB < 37 weeks were CCI (OR: 2.55; 95% CI: 1.28–5.10; *p* = 0.008) and history of sPTB (OR: 3.73; 95% CI: 1.87–7.44; *p* < 0.001). For sPTB < 34 weeks, the two independent variables were CCI below the 10th percentile (OR: 4.87; 95% CI: 1.42–16.63; *p* = 0.012), and history of sPTB (OR: 3.94; 95% CI:1.03–15.09; *p* = 0.044). When measuring the performance of the different covariates for the prediction of sPTB, the best AUC was obtained by CCI (0.62; 95% CI: 0.48–0.76). There was no significant association between sPTB and CL (Table 4).

### 3.5. Intraobserver and Interobserver Agreement of the Cervical Consistency Index

**Figure 3:**
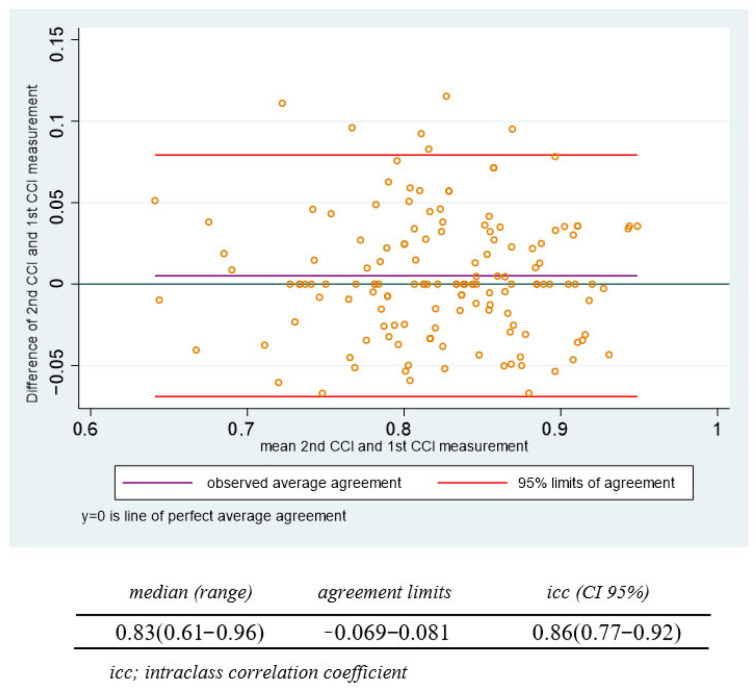
Intraobserver agreement of the cervical consistency index.

**Figure 4:**
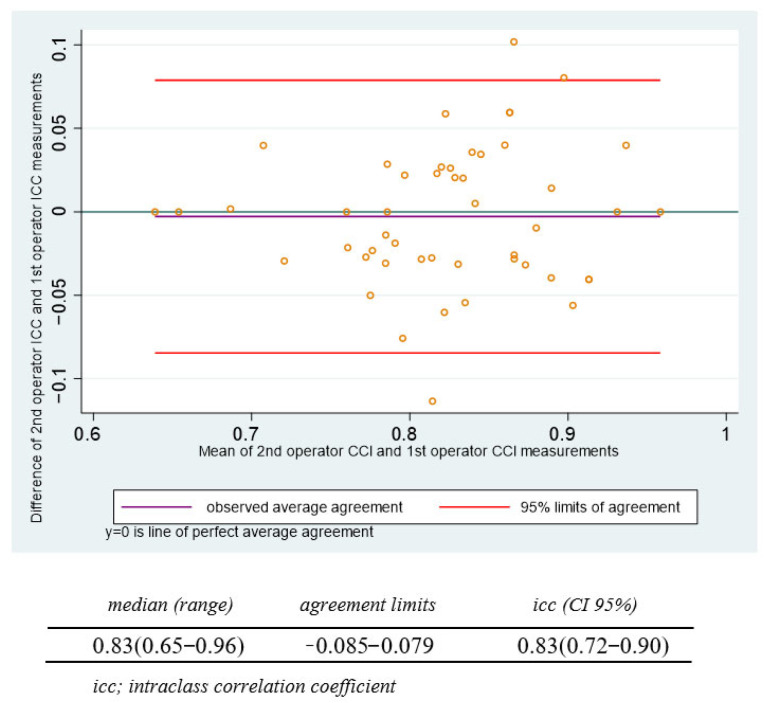
Interobserver agreement of the cervical consistency index.

Two measurements were evaluated in 109 cases by the same operator to establish the intraobserver variability, and two operators evaluated 49 patients to establish the interobserver variability. The relationship between the differences and means of the intraobserver and interobserver measurements are represented in the Bland–Altman plots (Figure 3 and Figure 4). The intraobserver ICC was 0.862 (95% CI, 0.769–0.920), and the interobserver ICC was 0.833 (95% CI, 0.722–0.902).

The Bland–Altman plot shows the magnitude of differences for the intraobserver CCI measurements.

The Bland–Altman plot shows the magnitude of differences for the interobserver CCI measurements.

## 4. Discussion

There are two main findings from the study. This first is that first-trimester CCI below the 10th percentile (CCI < 0.74) demonstrated a significant association with sPTB before 37 weeks, exhibiting 20% sensitivity and 90% specificity. The same percentile had a 33% sensitivity and 90% specificity for predicting sPTB before 34 weeks. The second finding is that women with a history of PTB who delivered before 34 weeks showed the highest sensitivity (67%) for CCI < 0.74, while the overall best predictor for sPTB was CCI, with an area under the curve (AUC) of 0.62. Also, we found a robust intraobserver and interobserver agreement, affirming the reliability of CCI measurements in predicting preterm birth. These results are in line with our previous observations, which other researchers have confirmed: the cervix softens before it shortens [16,17].

Many studies have evaluated CL performance early in pregnancy to select the population at risk of sPTB [18,19,20,21,22,23,24,25,26]. In the Conoscenti study [18], the authors evaluated the role of CL in the early second trimester (13–15 weeks) to predict sPTB; they did not find differences between groups. Carvalho et al. [19] evaluated CL at two points in the pregnancy; no differences were found between groups at 11–14 weeks. However, when comparing the outcome with CL at 22–24 weeks, the association was significant (39.3 mm vs. 26.7 mm, *p* = 0.0001), concluding that the cervix shortens more rapidly between the first and the second trimester in those patients who deliver prematurely. In the Berghella study [20], in pregnant women with a high risk of preterm birth, a short cervix (CL < 25 mm) at 10–14 weeks identified 14% of women who delivered preterm. Antsaklis et al. [21] used 27 mm and 30 mm as cut-offs at 11–14 weeks to predict PTB; the authors did not find predictive values for sPTB < 35 weeks and the predictive value for sPTB before 37 weeks (AUC 0.60; 95% CI 0.54–0.66, *p* = 0.001). Other studies arguing that the CL measurement technique may influence the results have reported an association between the first-trimester short cervix and sPTB. Souka et al. [22] evaluated the predictive value of a model including maternal characteristics and CL at 11–13 weeks; they did not include the uterine isthmus in the measurement. A cut-off of 27 mm identified 25% (3/12) of the patients who presented a cervix < 15 mm at 20–24 weeks. Greco et al. [23], measuring the CL at 11–13 weeks, found significant differences between patients who delivered preterm compared to patients who delivered at term (27,5 mm vs. 32.5 mm *p* < 0.0001); the prediction was not evaluated. Souka et al. [24] reported that median CL at 11–13 weeks was significantly shorter in the women who subsequently delivered preterm; CL predicted PTB before 37 weeks (OR 0.90; 95% CI, 0.522–0.671; AUC 0.596) and before 34 weeks (OR, 0.74; 95% CI, 0.649–0.869; AUC 0.759). The sensitivity for predicting 37 weeks was 27% for a fixed 25% screen positive rate. Recently, Feng et al. [25] found that CL was significantly shorter in women who delivered < 34 weeks compared to women who delivered at term (*p* < 0.001) with the two-line method, following the curvature of the cervical canal (AUC 0.658; 95% CI 0.637–0.677). In our study, the uterine isthmus was excluded in the CL measurement, and we did not find differences between cervical length in women who delivered at term 35 mm (33–38) and women who delivered preterm 35 mm (33–37) *p* = 0.845.

Regarding the other cervical characteristic considered, such as softness for the prediction of PTB, many techniques have been tested at different gestational ages; the characteristics of each are reviewed in detail by Feltovich et al. [26]. The author discusses the techniques’ limitations, and finally emphasizes the necessity of integrating quantitative techniques. Shear wave elastography (SWE) is one of the most evaluated. Hernandez-Andrade et al. [27] found that a soft cervix evaluated by SWE between 18 to 24 weeks of gestation increased the risk of sPTB < 37 weeks and <34 weeks. A soft cervix defined as an SWE at the internal OS < 25th percentile for the gestational age is a risk factor for sPTB < 34 weeks (OR 7.7; 95% CI 1.8–29.6) and for sPTD < 37 weeks (OR 4.4; 95% CI 1.4–12.0), independent of cervical length.

For the first trimester, Feng et al. [25] explored the potential value for the SWE to predict sPTB; the mean cervical SWE scores were significantly lower in women who delivered < 37 weeks (28.0 kPa vs. 30.6 kPa, *p* < 0.05). Women with a mean cervical SWE MoM <10th percentile had a RR of 2.42 (95% CI 1.29–4.55) and 7.81 (95% CI 2.13–28.60) for spontaneous delivery at <37 weeks and <34 weeks of gestation, respectively. The detection rate was 20.4% and 44.4% for sPTB at 37 and 34 weeks, respectively. In our study, the CCI showed a detection rate of 20% for sPTB < 37 weeks and 33% for sPTB < 34 weeks.

Taking all these results together, we consider, for the case of first-trimester sPTB prediction, that cervical softening is the characteristic that identifies early changes in the course of the disease, and the CCI is an efficient technique for detecting premature softening. Early intervention strategies, such as pharmacologic interventions (progesterone), targeted monitoring, and personalized care plans, can be implemented for women identified with a low CCI, potentially reducing the incidence of PTB and improving maternal and neonatal outcomes. Studies in larger populations are required to validate these findings.

We acknowledge that the first trimester CCI has limited ability to identify patients at risk of preterm birth, and considering the multiple pathways that cause preterm birth, it is unlikely that a single test will achieve better figures. Therefore, it will be necessary to add other measurements and biomarkers to build a model that improves its predictive capacity.

The study’s strengths lie in its prospective nature, the operator blindness for the cervical consistency index result, and the fact that the research team did not make clinical or therapeutic decisions based on the cervical measurements; additionally, the gestational age based on the CRL adds robustness to our findings. Reproducibility challenges were minimized by our approach, considering the cervical tissue’s biomechanical characteristics.

We recognize that the study has limitations, including a low prevalence of sPTB before 34 weeks, which impacted precision. We must be cautious with the sensitivity to identify patients with a history of preterm birth (67%) based on three cases. Additionally, a slight loss of participants to follow-up was observed, which affected accuracy; however, the study’s prospective design, the operator blinding, and the diverse participant pool from multiple institutions ensured a rigorous methodology and enhanced its credibility.

## 5. Conclusions

This study establishes the CCI as a promising and early predictive marker for sPTB risk, particularly before 34 weeks of gestation. This manuscript shows that a CCI below the 10th percentile in the first trimester significantly correlates with an increased likelihood of preterm birth. This research re-opens the question of first-trimester research for the prediction of sPTB.

## Figures and Tables

**Table 1:** Characteristics of the included population by groups of PTB < 37 weeks.

Characteristic	Term Birth *n* = 606	sPTB < 37 Weeks *n* = 61	*p*-Value
Maternal age (years) *	28 (24–32)	27(25–32)	0.118
Preterm birth history	41 (6.8%)	13 (21.3%)	<0.001
Smoking history	70 (11.5%)	4 (6.6%)	0.244
Body mass index *	25.3 (22.6–28.1)	26.5 (23.4–29.7)	0.224
GA at scan (weeks) *	13.1(12.5–13.5)	13.2 (12.5–13.5)	0.671
Cervical length (mm) *	35 (35–38)	35 (33–37)	0.845
CCI *	0.83 (0.78–0.87)	0.80 (0.76–0.85)	0.003
GA at delivery(weeks) *	38.6 (38–39.4)	36.2(34.5–36.4)	<0.001
Without previous pregnancies	223 (37%)	16 (26.2%)	0.097
Health care system Subsidized Contributive Special Not in the system	78 (28.2%) 172 (62.1%) 16 (5.8%) 11 (3.1%)	10 (29.4%) 17 (50.0%) 5 (14.7%) 2 (5.9%)	0.537 0.146 0.677
Marital status Married Live with partner Single	189 (31.2%) 334 (55.1%) 83 (13.7%)	13(21.3%) 44 (72.1%) 4 (6.5%)	0.480 0.544
Residence place Metropolitan area Outside	460 (76.0%) 145(24.0%)	42(68.8%) 19 (31.2%)	0.217
Nationality Colombian Venezuelan	576 (95.1%) 30 (4.9%)	58 (95.1%) 3 (4.9%)	0.991

The control group consisted of pregnant women who delivery at term. * Median (IQR); sPTB: spontaneous preterm birth; CCI: cervical consistency index; GA: gestational age.

**Table 2:** Predictive performance of the CCI for the prediction of sPTB.

Cut-Off	Sensitivity % (*n*/*N*)	Specificity % (*n*/*N*)	PPV % (*n*/*N*)	NPV % (*n*/*N*)	LR+ 95% CI	LR− 95% CI
sPTB < 37 weeks						
CCI (centile)						
0.71 (5th)	9.8 (6/61)	95.4 (576/604)	17.6 (6/34)	76.2 (576/631)	2.12 (0.91–4.92)	0.95 (0.85–1.06)
0.74 (10th)	19.7 (12/61)	90.4 (546/604)	17.1 (12/70)	91.8(546/595)	2.05 (1.17–3.60)	0.89 (0.77–1.03)
0.76 (15th)	24.6 (15/61)	85.9 (519/604)	15.0(15/100)	91.9 (519/565)	1.75 (1.08–2.83)	0.88 (0.75–1.03)
CCI < 0.70 *	9.8 (6/34)	96.2 (581/604)	20.7 (23/29)	91.4 (581/636)	3.40 (1.09–6.10)	0.87 (0.84–1.05)
sPTB < 34 weeks						
CCI (centile)						
0.71 (5th)	16.7 (2/12)	95.1 (621/653)	5.9 (2/34)	98.4 (621/631)	3.40 (0.92–12.60)	0.88 (0.67–1.14)
0.74 (10th)	33.3 (4/12)	90.0 (588/653)	5.8 (4/69)	98.7 (588/596)	3.35 (1.46–7.70)	0.74 (0.49–1.11)
0.76 (15th)	50.0 (6/12)	85.6 (559/653)	6.0 (6/100)	98.9 (559/565)	3.47 (1.91–6.30)	0.58 (0.33–1.03)
CCI < 0.69 *	16.7 (2/12)	96.3 (629/653)	7.7(2/26)	98.4 (629/639)	4.53 (1.21–17.06)	0.87 (0.66–1.13)

* the best cut-off point.

**Table 3:** Performance of CCI for the prediction of sPTB according to history of preterm birth.

No History of sPTB	Sensitivity % (*n*/*N*)	Specificity % (*n*/*N*)	PPV % (*n*/*N*)	NPV % (*n*/*N*)	LR+	LR−
sPTB < 37 weeks						
CCI at 10th percentile	15 (7/48)	91 (516/565)	13/7/49)	93 (516/557)	1.68	0.94
sPTB < 34 weeks						
CCI at 10th percentile	22 (2/9)	91 (550/604)	4 (2/56)	99 (550/557)	2.49	0.85
History of sPTB						
sPTB < 37 weeks						
CCI at 10th percentile	39 (5/13)	90 (37/41)	56 (5/9)	82 (37/45)	3.94	0.68
sPTB < 34 weeks						
CCI at 10th percentile	67 (2/3)	82 (44/51)	22 (2/9)	98 (44/45)	4.86	0.40

**Table 4:** Association between sPTB, CCI, cervical length, and history of PTB.

PTB < 37 Weeks	OR	5% CI	95% CI	*p*-Value	AUC
CCI <10th percentile	2.55	1.28	5.10	0.008	0.55 (0.50–0.60)
Cervical Length	1.00	0.95	1.06	0.843	0.50 (0.43–0.58)
History of PTB	3.73	1.87	7.44	<0.001	0.57 (0.52–0.62)
PTB < 34 weeks					
CCI < 10th percentile	4.87	1.42	16.63	0.012	0.62 (0.48–0.76)
Cervical Length	0.95	0.83	1.08	0.475	0.41 (0.22–0.60)
History of PTB	3.94	1.03	15.09	0.044	0.58 (0.45–0.71)

## Data Availability

The data that support the findings of this study are available on request from the corresponding author.

## Supplementary Materials

The following supporting information can be downloaded at: https://www.mdpi.com/article/10.3390/jcm13133906/s1, Table S1: Demographic and clinical characteristics of the entire cohort (*n* = 667).

## References

[1] Walani S.R. (2020). Global burden of preterm birth. Int. J. Gynaecol. Obstet..

[2] March of Dimes, pmNch, Save the Children, WHO. Born Too Soon: The Global Action Report on Preterm Birth. eds cp howson, mV Kinney, Je lawn. World Health Organization. Geneva. 2012. https://apps.who.int/iris/bitstream/handle/10665/44864/9789241503433_eng.pdf;jsessionid=C221360BA148E228A7A079C1EE4261E8?sequence=1.

[3] De Costa A., Moller A.B., Blencowe H., Johansson E.W., Hussain-Alkhateeb L., Ohuma E.O., Okwaraji Y.B., Cresswell J., Requejo J.H., Bahl R. (2021). Study protocol for WHO and UNICEF estimates of global, regional, and national preterm birth rates for 2010 to 2019. PLoS ONE.

[4] Blencowe H., Cousens S., Oestergaard M.Z., Chou D., Moller A.B., Narwal R., Adler A., Vera Garcia C., Rohde S., Say L. (2012). National, regional, and worldwide estimates of preterm birth rates in the year 2010 with time trends since 1990 for selected countries: A systematic analysis and implications. Lancet.

[5] Liu L., Oza S., Hogan D., Chu Y., Perin J., Zhu J., Lawn J.E., Cousens S., Mathers C., Black R.E. (2016). Global, regional, and national causes of under-5 mortality in 2000–15: An updated systematic analysis with implications for the Sustainable Development Goals. Lancet.

[6] Celik E., To M., Gajewska K., Smith G.C., Nicolaides K.H., Fetal Medicine Foundation Second Trimester Screening Group (2008). Cervical length and obstetric history predict spontaneous preterm birth: Development and validation of a model to provide individualized risk assessment. Ultrasound Obstet. Gynecol..

[7] Gudicha D.W., Romero R., Kabiri D., Hernandez-Andrade E., Pacora P., Erez O., Kusanovic J.P., Jung E., Paredes C., Berry S.M. (2021). Personalized assessment of cervical length improves prediction of spontaneous preterm birth: A standard and a percentile calculator. Am. J. Obstet. Gynecol..

[8] Parra-Saavedra M., Gómez L., Barrero A., Parra G., Vergara F., Navarro E. (2011). Prediction of preterm birth using the cervical consistency index. Ultrasound Obstet. Gynecol..

[9] Baños N., Murillo-Bravo C., Julià C., Migliorelli F., Perez-Moreno A., Ríos J., Gratacós E., Valentin L., Palacio M. (2018). Mid-trimester sonographic cervical consistency index to predict spontaneous preterm birth in a low-risk population. Ultrasound Obstet. Gynecol..

[10] Baños N., Julià C., Lorente N., Ferrero S., Cobo T., Gratacos E., Palacio M. (2018). Mid-Trimester Cervical Consistency Index and Cervical Length to Predict Spontaneous Preterm Birth in a High-Risk Population. AJP Rep..

[11] van der Merwe J., Couck I., Russo F., Burgos-Artizzu X.P., Deprest J., Palacio M., Lewi L. (2020). The Predictive Value of the Cervical Consistency Index to Predict Spontaneous Preterm Birth in Asymptomatic Twin Pregnancies at the Second-Trimester Ultrasound Scan: A Prospective Cohort Study. J. Clin. Med..

[12] Wharton L.K., Anumba D.O.C. (2023). Techniques for detecting cervical remodeling as a predictor for spontaneous preterm birth: Current evidence and future research avenues in patients with multiple pregnancies. J. Matern. Fetal Neonatal Med..

[13] Sonek J., Shellhaas C. (1998). Cervical sonography: A review. Ultrasound Obstet. Gynecol..

[14] Becerra-Mojica C.H., Parra-Saavedra M.A., Diaz-Martinez L.A., Martinez-Portilla R.J., Rincon Orozco B. (2022). Cohort profile: Colombian Cohort for the Early Prediction of Preterm Birth (COLPRET): Early prediction of preterm birth based on personal medical history, clinical characteristics, vaginal microbiome, biophysical characteristics of the cervix and maternal serum biochemical markers. BMJ Open.

[15] Bland J.M., Altman D.G. (1986). Statistical methods for assessing agreement between two methods of clinical measurement. Lancet.

[16] Torres J., Faris I., Callejas A. (2019). Histobiomechanical Remodeling of the Cervix during Pregnancy: Proposed Framework. Math. Probl. Eng..

[17] McFarlin B.L., Bigelow T.A., Laybed Y., O’Brien W.D., Oelze M.L., Abramowicz J.S. (2010). Ultrasonic attenuation estimation of the pregnant cervix: A preliminary report. Ultrasound Obstet. Gynecol..

[18] Conoscenti G., Meir Y.J., D’Ottavio G., Rustico M.A., Pinzano R., Fischer-Tamaro L., Stampalija T., Natale R., Maso G., Mandruzzato G. (2003). Does cervical length at 13–15 weeks’ gestation predict preterm delivery in an unselected population?. Ultrasound Obstet. Gynecol..

[19] Carvalho M.H., Bittar R.E., Brizot M.L., Maganha P.P., Borges da Fonseca E.S., Zugaib M. (2003). Cervical length at 11–14 weeks’ and 22-24 weeks’ gestation evaluated by transvaginal sonography, and gestational age at delivery. Ultrasound Obstet. Gynecol..

[20] Berghella V., Talucci M., Desai A. (2003). Does transvaginal sonographic measurement of cervical length before 14 weeks predict preterm delivery in high-risk pregnancies?. Ultrasound Obstet. Gynecol..

[21] Antsaklis P., Daskalakis G., Pilalis A., Papantoniou N., Mesogitis S., Antsaklis A. (2011). The role of cervical length measurement at 11–14 weeks for the prediction of preterm delivery. J. Matern. Fetal Neonatal Med..

[22] Souka A.P., Papastefanou I., Michalitsi V., Papadopoulos G.K., Kassanos D. (2011). A predictive model of short cervix at 20–24 weeks using first-trimester cervical length measurement and maternal history. Prenat. Diagn..

[23] Greco E., Lange A., Ushakov F., Calvo J.R., Nicolaides K.H. (2011). Prediction of spontaneous preterm delivery from endocervical length at 11 to 13 weeks. Prenat. Diagn..

[24] Souka A.P., Papastefanou I., Michalitsi V., Salambasis K., Chrelias C., Salamalekis G., Kassanos D. (2011). Cervical length changes from the first to second trimester of pregnancy, and prediction of preterm birth by first-trimester sonographic cervical measurement. J. Ultrasound Med..

[25] Feng Q., Chaemsaithong P., Duan H., Ju X., Appiah K., Shen L., Wang X., Tai Y., Leung T.Y., Poon L.C. (2022). Screening for spontaneous preterm birth by cervical length and shear-wave elastography in the first trimester of pregnancy. Am. J. Obstet. Gynecol..

[26] Feltovich H., Carlson L. (2017). New techniques in evaluation of the cervix. Semin. Perinatol..

[27] Hernandez-Andrade E., Maymon E., Luewan S., Bhatti G., Mehrmohammadi M., Erez O., Pacora P., Done B., Hassan S.S., Romero R. (2018). A soft cervix, categorized by shear-wave elastography, in women with short or with normal cervical length at 18–24 weeks is associated with a higher prevalence of spontaneous preterm delivery. J. Perinat. Med..

