# Supplementary material for: Performance of the First-Trimester Cervical Consistency Index to Predict Preterm Birth
Source: J Clin Med. 2024 Jul 3;13(13):3906. doi: 10.3390/jcm13133906 (PMC11242471; doi:10.3390/jcm13133906)
Supplement: Supplementary file 1 [file jcm-13-03906-s001.zip › jcm-3021103-supplementary.pdf]

Table S1. Demographic and clinical characteristics of the entire cohort (n=667)

Characteristic	n	%
**Age** (years) Median (IQR)	28 (24-32)	
**Recruitment center**		
HUS	98	14.7
FOSCAL	569	85.3
**Marital status**		
Married	202	30.3
Single	87	13.0
Live with a partner	378	56.7
**Place of residency**		
Metropolitan area	502	75.3
Outside	165	24.7
**Health care**		
Subsidized	167	25.0
Contributive	458	68.7
Special	29	4.3
Uncovered	13	2.0
**Labor**		
Employee	289	43.3
Freelance	119	17.8
Home	220	33.0
Student	37	5.6
Unemployed	2	0.3
**Pregnancy number**		
0	239	35.8
1	269	40.4
2	97	14.5
3 +	62	9.3
**Preterm birth history**		
Yes	54	8.1
No	373	56.0
First pregnancy	239	35.9
**Pregnancy diseases**		
Diabetes Mellitus	18	2.7
Hypothyroidism	35	5.2
HTA in pregnancy	23	3.5
Others	27	4.0
No diseases	564	84.6
**Smoking history**	74	11.99
**Body Mass Index**		
Underweight	21	3.2
Normal	298	44.7
Overweight	227	34.0
Obesity	121	18.1
**Gestational age at scan** Median (IQR).	13.1 (12.5-13.5)	
**Cervical length** (mm) Median (IQR).	35 (33-38)	
**Cervical Consistency Index** Median (IQR).	0.83 (0.78-0.87)	
**Gestational age at delivery** Median and (IQR).	38.5 (37.6-39.4)	
